# Management of a Patient With Gynecomastia With Ventriculoperitoneal (VP) Shunt: A Case Report

**DOI:** 10.7759/cureus.102407

**Published:** 2026-01-27

**Authors:** Jayant Dash, Ipsa Mohapatra

**Affiliations:** 1 Department of Plastic and Reconstructive Surgery, Kalinga Institute of Medical Sciences, Bhubaneswar, IND; 2 Department of Community Medicine, Kalinga Institute of Medical Sciences, Bhubaneswar, IND

**Keywords:** breast, corrective surgery, gynecomastia, power assisted liposuction, ventriculoperitoneal shunt

## Abstract

Ventriculoperitoneal (VP) shunts traverse the anterior chest through breast parenchyma. Any breast surgery planned for patients with VP shunts requires extra caution, as it poses a risk of injury to the shunt. This is a case report of a 19-year-old male patient who had bilateral (B/L) gynecomastia, with a pre-existing VP shunt, and was operated successfully by power-assisted liposuction (PAL) without any displacement or injury to the shunt. This was possible only because of a collaborative, multidisciplinary approach to preserve the shunt; in this case, it involved an interprofessional discussion between the operating plastic surgeon, neurosurgeon, and radiologist during preoperative planning. The patient had a satisfactory recovery with good cosmesis postoperatively.

## Introduction

Ventriculoperitoneal (VP) shunts are tubes that connect the cerebral cavity to the peritoneal cavity, thereby draining the excess cerebrospinal fluid, in patients with intracranial hypertension and hydrocephalus [1,2]. A shunt is a one-way valve catheter connecting the ventricle to the peritoneum. It passes subcutaneously through the anterior chest, involving part of the breast parenchyma. This demands extra caution while performing any type of breast surgery because of the fear of injury to the shunt [3,4]. There is a case report of two operated cases, one from the USA and one from the UAE, of a female with gigantomastia [1], but none among male patients with gynecomastia. Due to the paucity and non-availability of literature describing this specific scenario, the authors have reported this case.

We are presenting a case of gynecomastia in a patient with a pre-existing VP shunt who underwent surgery for correction of gynecomastia. To ensure the safety of the patient and a good surgical outcome, the primary operating surgeon focused on preoperative planning with interdepartmental discussion and imaging technique.

## Case presentation

**Figure 1:**
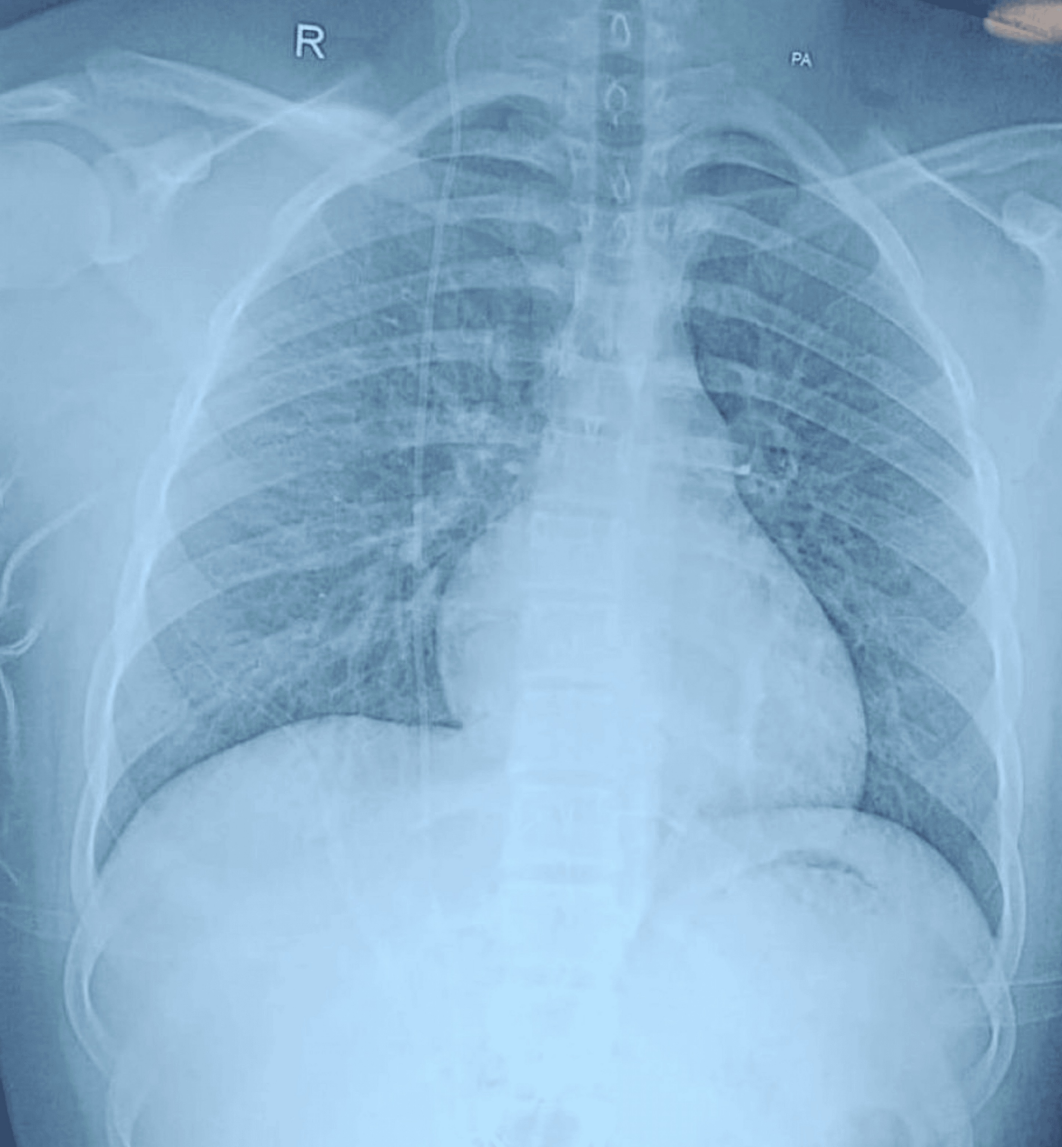
Pre-operative X-ray showing ventriculoperitoneal (VP) shunt position

A 19-year-old male presented to us with bilateral (B/L) gynecomastia, Simon Grade 2b. He was feeling awkward and had a history of body shaming because of B/L enlargement of his breasts for almost five years. The exact cause of gynecomastia was investigated by all necessary investigations, including hormonal assay. All reports were within normal limits, and the cause was found to be idiopathic. His past medical history revealed VP shunt insertion after brain tumor surgery nine years ago. There was no history of any shunt revision surgery in this case. At the time of presentation, he did not have any neurological symptoms. Preoperative discussion with the neurosurgery department was carried out for confirmation of shunt viability and intraoperative assistance if required. The chest X-ray of the patient was reviewed and discussed with the radiologist. The radiologist saw the baseline preoperative X-ray and was told that he would again be consulted in the postoperative period, in case there were any complications. The chest X-ray (PA view) to locate the course of the VP shunt through the chest is shown in Figure 1.

**Figure 2:**
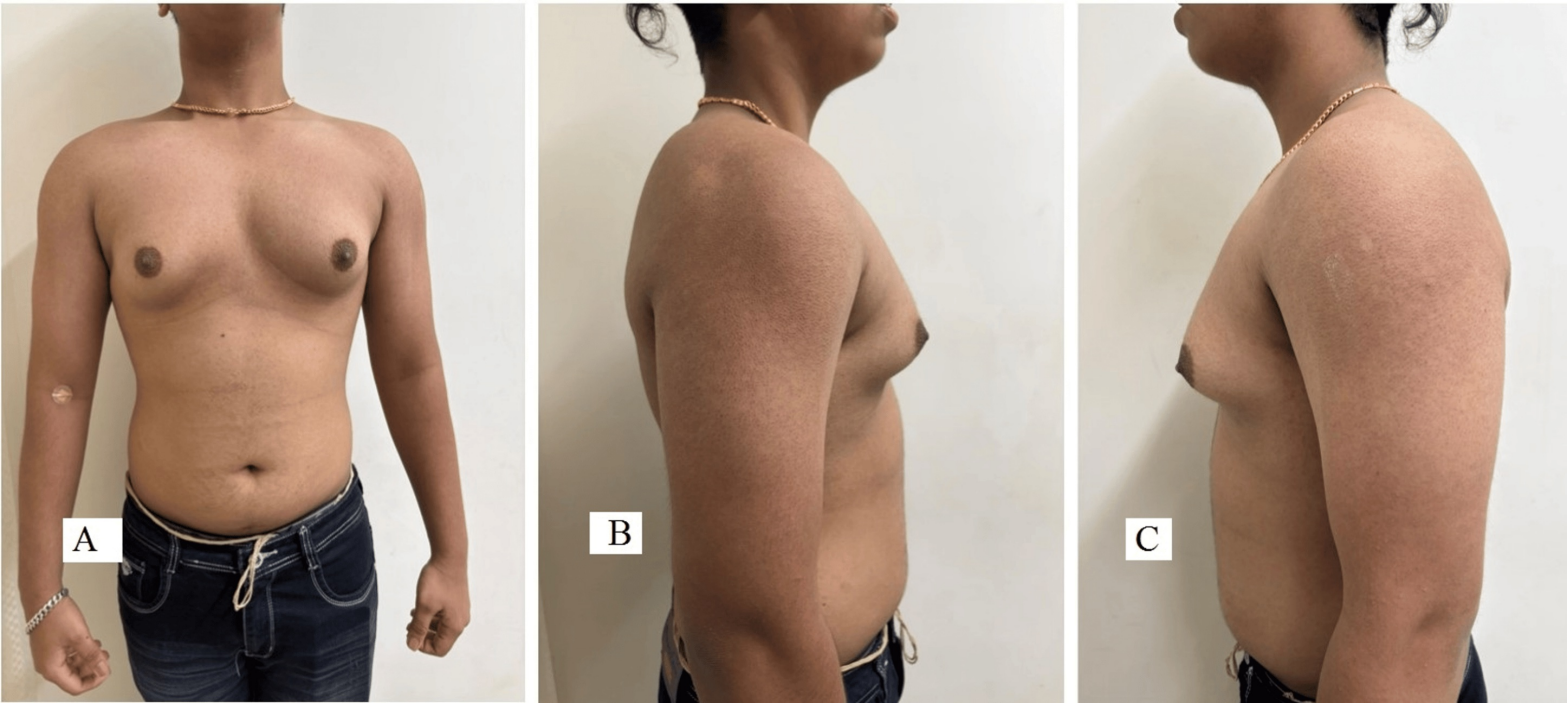
Preoperative pictures (A - anterior, B - right lateral, and C - left lateral) of the chest

The preoperative pictures of the patient, with various views, were taken before surgery, as shown in Figure 2.

**Figure 3:**
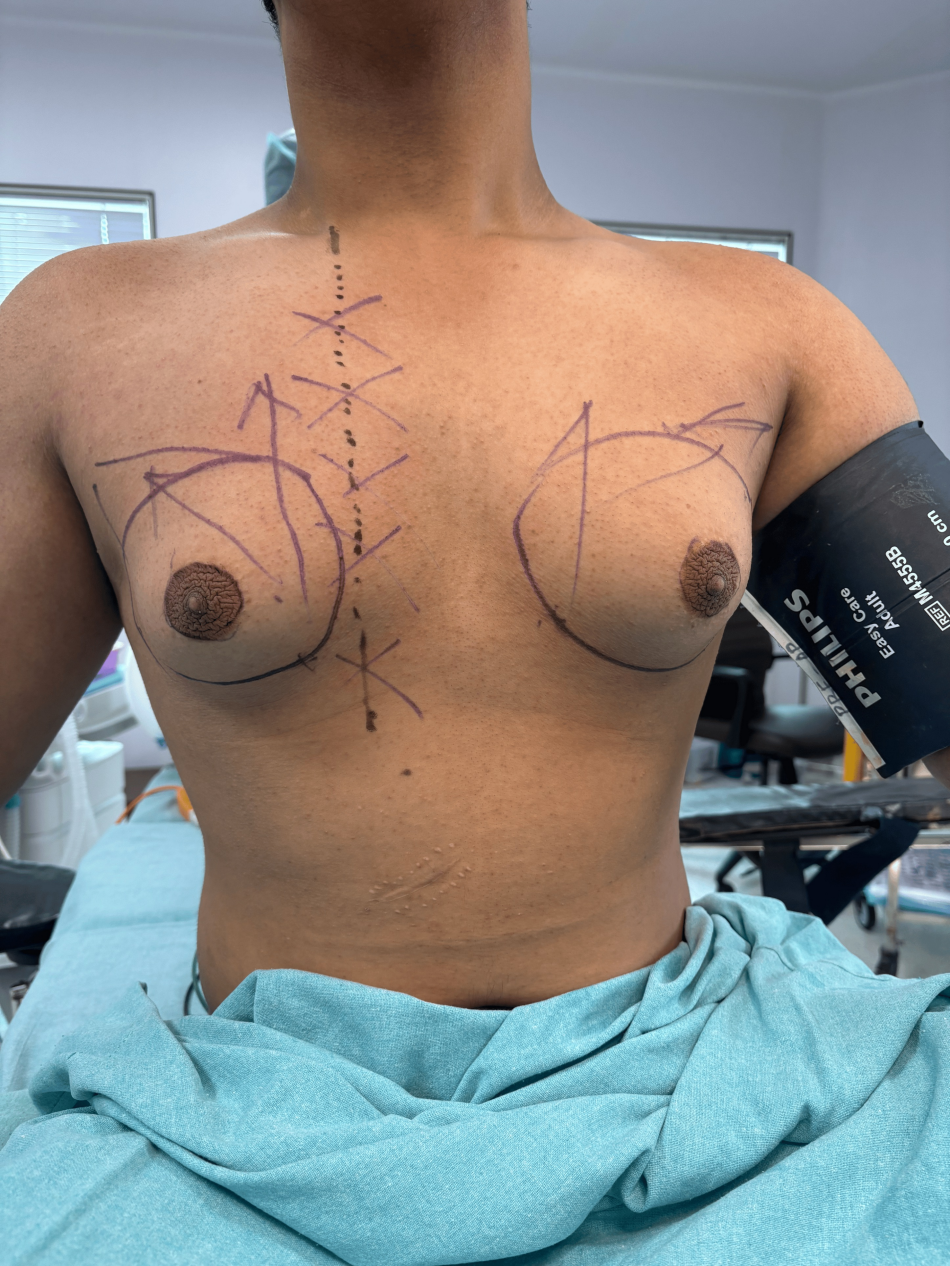
Pre-operative marking of the skin of the anterior chest wall

The entire path of the shunt was marked on the skin before antiseptic prepping and draping (Figure 3).

**Figure 4:**
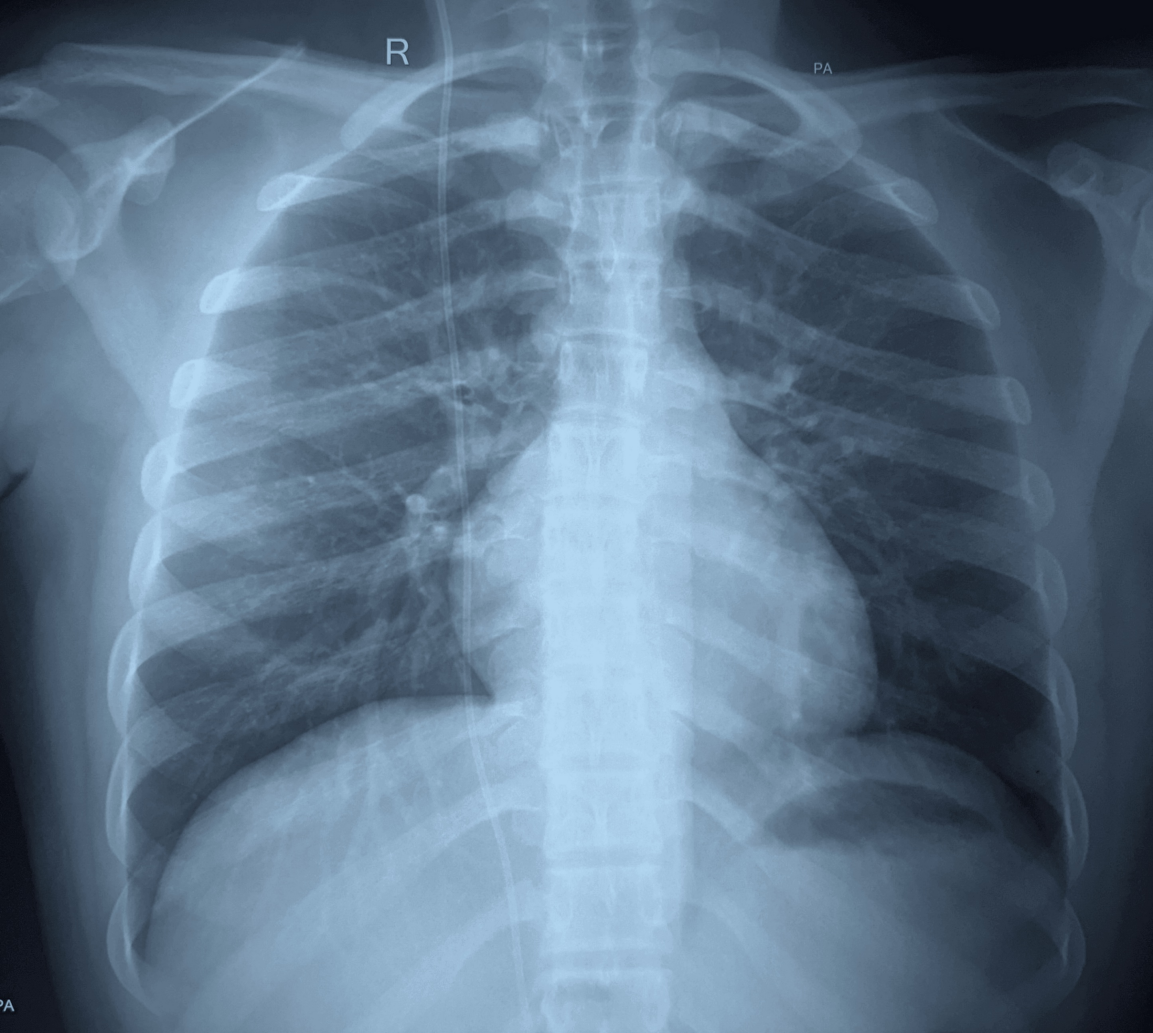
Immediate postoperative X-ray of chest (PA view) showing intact ventriculoperitoneal (VP) shunt position

Power-assisted liposuction (PAL) was done, adhering to standard surgical technique. Extra precaution was taken both during tumescent infiltration and liposuction by avoiding the area marked for the shunt. As a usual practice, liposuction is done from the lateral side to make the incision inconspicuous. In this case, it was done from the medial side to avoid shunt injury. If so, they should state it as a tip. A postoperative X-ray was done; this helped confirm that the pathway of the shunt was neither injured nor disturbed (Figure 4).

**Figure 5:**
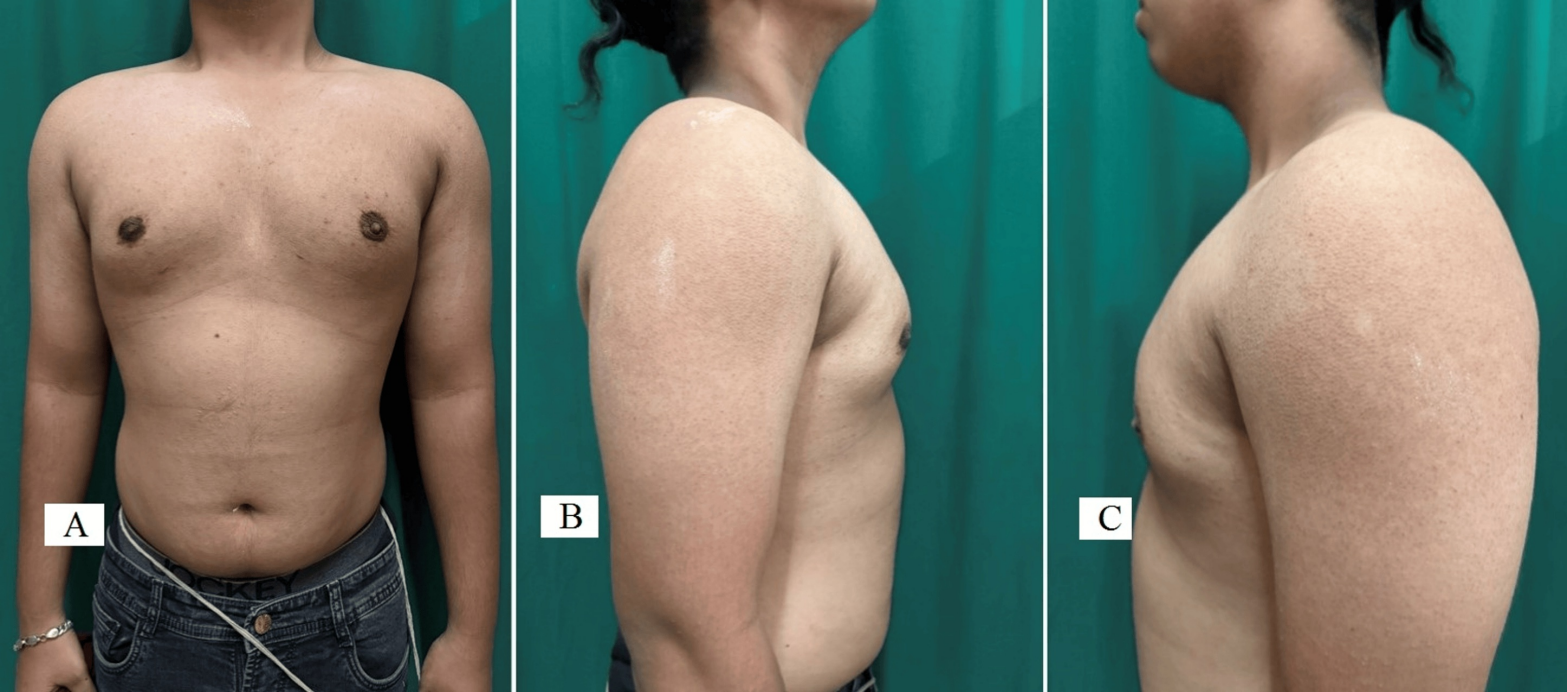
The three-month follow-up postoperative pictures (A - anterior, B - right lateral, and C - left lateral) of the chest

On follow-up, the patient's postoperative course was found to be uneventful. He was satisfied with the results and aesthetic look. The postoperative pictures of the patient were taken three months after surgery (Figure 5).

## Discussion

Patients with VP shunts have seen shunt failure, which increases with time. A follow-up study done to find long-term shunt survival over a period of four years found around 38% failure after one year, around 42% by the end of the second year, and 58% in four years [5]. It has been seen that the complications related to VP shunts were common, requiring multiple revision surgeries, often required throughout the life of the patient. This complication can further be increased if there is any surgery on the anterior chest wall, as the tubing traverses the same.

Breast surgeries in such patients with a preexisting VP shunt can have higher complications. A study by Crawford et al. [6] noted an intraoperative injury to the VP shunt at the time of mastectomy. The reason documented for this could be as a result of the right breast medial dissection mastectomy flap or due to manipulation of the medial pectoralis muscle during surgery [6]. In another article by Zawadiuk et al. [7], they reported that a patient with breast implant placement had a VP shunt fracture secondary to subglandular tissue expansion; this resulted in recurrent cerebrospinal fluid pseudo-cyst. Sometimes the manifesting symptoms may be difficult to correlate, as in this case, the presenting symptom was cerebrospinal fluid pseudo-cyst, while the cause was due to fracture of the VP shunt at the time of breast surgery for breast asymmetry. The symptoms were resolved after surgeries of both the implant and the shunt [7].

The current case was mainly concerned with the management of gynecomastia with an existing VP shunt. All precautions were taken to ensure a safe and optimal outcome during the procedure. The success of the surgery reiterates the fact that proper preoperative planning plays a crucial role. Planning in this case included imaging of the chest, which helped to delineate the course of the VP shunt. Conventional chest radiography, which was done to visualize the entire pathway of the shunt, helped assess for breaks, distal catheter migration, or disconnections; the result showed the VP shunt to be para-stermal. The idea of using imaging as a modality in preoperative management was decided after a literature review of the same [8]. An involvement of a neurosurgeon and consent for readiness to intervene, in case of need, during surgery were ensured. Furthermore, imaging (chest X-ray) was taken even after the surgery; the baseline and postoperative imaging were used for comparison of the pre-surgery and post-surgery; and this baseline image would further help in patient counseling in case a complication arose. The authors recommend that all patients with a similar case history should be counselled elaborately, in the preoperative period, to help them make an informed decision. Counselling will have a pivotal role and help them understand the benefits and risks of the procedure in such cases of gynecomastia, which have a preexisting VP shunt.

The limitation of this research reported could be that it is a single case finding. A case series of the same will help in generating more robust evidence and reaffirm the facts. The authors wish to continue this research in the future and publish a case series of the same.

## Conclusions

Gynecomastia surgeries in patients with VP shunts are associated with atypical complications, which are quite different in normal patients (without shunts). As these complications can involve both the shunt and the breast, preoperative planning and counselling have a pivotal role. Preoperative chest radiograph helped identify the position of the shunt, giving the surgeon an insight into the pathway of the shunt; this helped the surgeon to avoid any shunt damage. A collaborative, interdepartmental approach, along with good counselling, ensures an immediate corrective action if there is a dislodgment of the VP shunt. We recommend routine preoperative imaging and neurosurgical involvement in similar cases.
